# Normoxic low‐altitude simulation (at 714 mmHg) improves limb blood perfusion in mice with hindlimb ischemia

**DOI:** 10.14814/phy2.14228

**Published:** 2021-01-27

**Authors:** Anmol Shahid, Trevor H. Stenson, Michael S. Mcmurtry

**Affiliations:** ^1^ Vascular Biology Research Group Department of Medicine University of Alberta Edmonton Canada

**Keywords:** altitude hemodynamics, low‐altitude simulation, lowered barometric pressure, peripheral arterial disease

## Abstract

Humans have fewer cardiovascular events and improved outcomes after cardiovascular events when living at low and moderate altitudes (<3000 m) above sea level. We have previously shown that low‐altitude simulation using reductions in barometric pressure enhances vasodilation *ex vivo* in arterial segments and reduces systemic vascular resistance *in vivo* and can also improve left ventricular function after a myocardial infarction. We hypothesize that low‐altitude simulation could also improve hindlimb ischemia, a model of peripheral artery disease in humans. We performed femoral artery ligation to generate hindlimb ischemia in 3‐month‐old C57BL6 mice. Control group mice (*n* = 10) recovered at 754 mmHg (control) for 14 days. Treatment group mice (*n* = 15) were placed in a low‐altitude simulation chamber (at 714 mmHg) to recover from surgery for 3‐hours daily for 14 days. Hindlimb perfusion imaging using a laser Doppler line scanner was performed for all mice prior to the surgery, and then on days 1, 3, 7, and 14 post‐surgery. At 2 weeks, ischemic reserve was significantly higher in the treatment group mice (0.50 ± 0.13 vs. 0.20 ± 0.06; *p* = 0.01). Treatment mice had higher functional scores and were able to walk better at two weeks. There was approximately three times less HIF1α found via western blotting and a small but statistically significant improvement of lectin perfusion in calf tissue of treatment mice. We conclude that low‐altitude simulation improves blood perfusion in murine hindlimb ischemia. This approach may have therapeutic implications for humans with peripheral artery disease.

## Graphical abstract

Normoxic low‐altitude simulation (714 mmHg), when applied acutely, has the capacity to improve limb blood perfusion after femoral artery ligation in mice. This is a novel finding that illustrates the capacity of normoxic low‐altitude simulation to be use as a non‐invasive therapy in human patients of peripheral arterial disease.

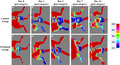

**Abbreviations**
HLI: hindlimb ischemia
MI: Myocardial infarction
PAD: Peripheral arterial disease

**New and Noteworthy**

We have previously found that low‐altitude simulation improved hemodynamics *in vivo*, with higher cardiac output and reduced systemic vascular resistance, and could be used in mice to improve cardiac blood perfusion after myocardial infarction. We now apply this model of improving blood flow in a mouse model of hindlimb ischemia. This finding could lead to a non‐invasive treatment to supplement current therapies for peripheral arterial disease.

## 1 INTRODUCTION

Atherosclerosis contributes to a myriad of cardiovascular diseases, including peripheral arterial disease (PAD), which is caused by atherosclerotic plaques in the leg arteries leading to arterial stenosis or occlusion (Hirsch, 2006). The resulting ischemia can be very severe, causing pain on rest and tissue loss through ulceration and gangrene (Morley et al., 2018). PAD is also associated with stroke, myocardial infarction (MI), vascular dementia, renovascular disease, and mesenteric disease (Fowkes et al., 2008; Morley et al., 2018). The Global Burden of disease study states that PAD was responsible for over 40,000 deaths in 2013 alone and patients with severe symptomatic large vessel PAD had a 25% chance of death from cardiovascular causes within a year (Fowkes et al., 2017). The 1‐year mortality of patients presenting with severe, chronic PAD can be up to 45% (Hiatt, 2001). However, a large percentage of PAD cases are not symptomatic, and many countries do not screen for PAD (Conte & Vale, 2018). Data from the NHANES study of 2174 persons in the United States showed that more than 95% of patients with PAD presented with one of more cardiovascular risk factors (Selvin & Erlinger, 2004) which include diabetes, hyperlipidemia, hypertension, metabolic syndrome, older age, and smoking status among others (Joosten et al., 2012). While these risk factors explain much of the risk for atherosclerosis leading to PAD, other exposures may modify the risk.

Several studies have reported associations between a higher altitude of residence and lower mortality from coronary artery disease, myocardial infarction, and stroke (Baibas et al., 2005; Fabsitz & Feinleib, 1980; Gordon et al., 1977; Voors & Johnson, 1979). A notable study of individuals living between 259 and 1960 m showed a risk reduction of −22% per 1000 m of altitude above sea level for MI and −12% per 1000 m for stroke (Faeh et al., 2009). Interestingly, others have reported negative consequences of altitude on cardiovascular health in humans with a history of previous cardiovascular events (Al Tahan et al., 1998; Al‐Huthi et al., 2006). There is some evidence that altitude exposure may alter vascular function. A 2001 study suggested that higher altitude residents exhibited a superior ability to increase blood flow velocity as a response to muscular ischemia, compared to lowland dwellers being conditioned to higher altitude. However, this study did not provide a sea level baseline for lowland dwellers, making it difficult to infer changes to the vascular function of the lowlanders reliably (Schneider et al., 2001). Another study presented that altitude exposure in lowlanders caused persistent impairment in vascular function, potentially mediated by oxidative stress and sympathoexcitation (Lewis et al., 2014). However, these studies were conducted at altitudes above 5000 m, a higher altitude than previous studies showing cardiovascular benefit from altitude. Although it seems that high altitude can have detrimental effects to vascular function, low and moderate altitudes above sea level are strongly linked with lower all‐cause mortality in the general US population (Winkelmayer et al., 2009). Despite the limitations of prior reports, there appears to be a strong correlation between low and moderate altitude of residence and protection from atherosclerotic cardiovascular disease. In our previous work with mouse models of MI, we showed that low‐altitude simulation treatment could be used to improve left ventricular function after MI (Shahid et al., 2019) by increasing blood perfusion to the ischemic area, leading us to believe altitude simulation may also be beneficial in therapeutic use for PAD.

Currently, PAD is managed with risk factor reduction and exercise is commonly prescribed to improve mildly symptomatic presentations of the disease (Hankey et al., 2006). For patients presenting with severe symptoms or tissue loss, revascularization is required through surgery or angioplasty. However, it is often difficult to achieve revascularization and amputation may become necessary, leading to disability and extensive costs to the health care system and the economy (Vartanian & Conte, 2015). Additionally, there are no effective medications for PAD (Kundhal et al., 2007), and therefore additional treatments to directly improve limb perfusion are needed. Based on our prior work showing improvement in left ventricular function post‐MI in mice on a regimen of daily low altitude exposure in an altitude simulation chamber (Shahid et al., 2016, 2019), we hypothesized low‐altitude simulation would improve ischemic limb perfusion in a murine model of hindlimb ischemia (HLI).

## 2 METHODS

### 2.1 Ethical approval

All protocols used in this study were approved by the University of Alberta Animal Policy and Welfare Committee (UAPWC) in accordance with the Canadian Council on Animal Care (CCAC) guidelines.

### 2.2 Surgical induction of hindlimb ischemia

Three‐month‐old male C57BL6 mice (Charles River; Wilmington, MA) were given access to standard chow and water and were housed on a 12 h–12 h light–dark cycle. Mice were housed as three animals per cage, and chow and water were always available to the animals, except for inside the altitude simulation chamber. Mice (*n* = 25) were intubated and anesthetized using 2.5% isofluorane on a heated surgical platform. A 3‐mm incision was made in the left inguinal area and the common femoral and superficial femoral arteries were identified tracing along the left thigh. Two 5.0 polypropylene sutures were placed around the left superficial femoral artery tightly, taking care not to injure the adjacent vein or nerve. The suture was tightened to completely and permanently restrict blood flow through the femoral artery. The wound was closed using 5.0 prolene interrupted suture.

The animals were split into two groups, a control group (*n* = 10) and a treatment group (*n* = 15). Immediately after the surgery was completed, animals in the experimental group were placed in an altitude simulation chamber with a barometric pressure of 714 mmHg for a period of 3 hours to simulate low altitude before continuing recovery at room barometric pressure. We have previously shown small reductions in barometric pressure to have an acute and immediate effect on vascular function *ex vivo* and cardiovascular hemodynamics *in vivo* and we chose treatment with 714 mmHg at 3 h daily in light of our previous work (Shahid et al., 2016, 2019; Shahid, Patel, et al., 2016). As the animals did not have access to food and water inside the altitude simulation chamber due to space limitations, three hours was deemed to be the maximum amount of time the animals could ethically be kept in the chamber by the Animal Care and Use Committee at the University of Alberta. Furthermore, we predicted three hours to be enough to exert a lasting therapeutic effect as we previously showed immediate increases in tissue perfusion with application of low‐altitude simulation (Shahid et al., 2019). Control group animals recovered at room barometric pressure (754 mmHg) following the surgery. The experimental group of animals was administered altitude simulation treatment (at 714 mmHg) daily for two weeks (14 days), for 3 hours per day. All animals were monitored twice daily to ensure the animals recovered as expected post‐operatively. As per recommendation of the Veterinarian with the University of Alberta Animal Care and Use Committee, Buprenorphine was used as an analgesic. After 14 days, the animals were sacrificed using cervical dislocation and calf tissue was harvested.

We did not collect any metrics on the eating and drinking habits of these mice as we aimed to mimic a low altitude of 523 m above sea level (714 mmHg), and any significant changes to appetite at low altitudes have not previously been reported. Furthermore, there were no significant differences in the weight of control and treatment group mice.

### 2.3 Low‐altitude simulation

For normoxic low‐altitude simulation treatment, animals were placed in a specially constructed cylindrical 20.1 × 26.67 cm chamber (Figure S1; https://doi.org/10.6084/m9.figshare.7859972.v2) that could be sealed to maintain a given barometric pressure. A baseline pressure was noted with a pressure sensor relative to the barometric pressure denoted by the meteorological service of Canada (Edmonton, Alberta). With the experimental animals enclosed in the chamber, low‐altitude simulation corresponding to a pressure of 714 mmHg (approximately 40 mmHg below the barometric pressure of the laboratory room) was maintained through a vacuum controller (Buchi V‐850; New Castle, DE). This pressure was chosen carefully to simulate low altitude without inducing the effects of hypoxia in the mice.

### 2.4 Laser Doppler imaging and analysis

Imaging to discern changes in blood perfusion were captured using a laser Doppler line scanner, MoorLDLS2‐IR (Moore Instruments). Animals were anesthetized using 2.5% isoflurane and placed on a heated matt (37°C) for 5 minutes. The laser Doppler line scanner was set to high acquire high resolution images and calibrated at 14 cm from the subject. A depilatory cream was used to remove the fur from both lower limbs thoroughly. Laser Doppler images for the lower body (both limbs) were acquired immediately prior to and 5 min after the completion of the femoral ligation procedure. Laser Doppler images were also acquired on Days 3, 7, and 14 after the surgery. Care was taken to remove any hair regrowth before each imaging session. It was ensured that ambient light in the room was consistent throughout all imaging sessions. Images were analyzed using the software accompanying the MOOR LDLS laser Doppler scanner. For each image acquired, a flux image was generated showing a gradient of color from blue to red, the latter denoting a higher degree of blood perfusion. A region of interest was selected in each limb (control vs. experimental) and flux values were generated. These values were standardized to the leg that did not receive surgical intervention.

### 2.5 Functional scoring

We assigned Tarlov scores (Brenes et al., 20122012; Tarlov, 1954) (as shown in Table 1) to evaluate any functional deficits in the control and treatment groups of mice after femoral artery ligation surgery was performed. Tarlov scoring was completed on days 7 & 14 following the femoral artery ligation surgery.

**TABLE 1:** Tarlov scores used to assess walking ability after femoral artery ligation surgery. Adapted from Brenes et al. (2012)

Tarlov score	Function
0	No movement
1	Barely perceptible movement, no weight bearing
2	Frequent and vigorous movement, no weight bearing
3	Supports weight, may take 1 or 2 steps
4	Walks with only mild deficit
5	Normal but slow walking
6	Full and fast walking

### 2.6 Lectin perfusion imaging and analysis

We used an EVOS FL LED fluorescence microscope (Invitrogen) to complete the imaging. Mice were injected with 5 mg of lectin fluorescein ricinus communis agglutinin I (Vector Laboratories Inc) using a central venous cannula for 5 minutes prior to sacrifice, calf tissue isolation, and flash freezing. We sliced the tissues at 20‐μm thick and fixed them with 4% paraformaldehyde before imaging. We used the Image J (National Institutes of Health, MA, USA) software to semiquantify regions of interest were in arbitrary fluorescence units. For quantification, we excluded the background from measured green fluorescence units with background correction using a “rolling ball” method with a radius of 50.0 pixels. Image J was used to retrieve the RGB (red, blue, green) profile of the selected area, providing the intensity of each color in the region. Lectin signal was imaged in the green channel, and the green intensity signal in the ROI (region of interest) was recorded for comparison of lectin signal between the control samples and treatment tissue samples.

### 2.7 Western blot analysis

We performed western blotting with standard technique using 25 μg of protein per sample obtained from calf tissue of the ischemic limbs of select mice from the control (*n* = 5) and treatment groups (*n* = 8). We used a HIF1α antibody (cat. no#3716S) from Cell Signaling, Technology Inc of Beverly, MA, USA at a suggested 1:1000 dilution. We normalized the protein expression to actin to correct for variablility in the amount of protein loaded in each lane before developing and quantifying the films using the densitometry technique in the Image J program (National Institutes of Health, MA, USA). The ImageJ “Gel Analysis” function was utilized to generate numerical values for each band that could then be quantified.

### 2.8 Statistical analysis

In this study, we aimed for a power of 80%, expecting at least a 10% difference in laser Doppler flux values between the control group and the treatment group and found that a sample size of 10–15 animals per group would be sufficient. All data are presented as mean ± SEM. We used a one‐way ANOVA to compare the ischemic reserve values generated from repeated laser Doppler imaging of the control and treatment group of mice. Multiple comparisons were made using the Tukey's post hoc test. The unpaired t‐test was used for comparison between control and treatment groups where appropriate and *p* < 0.05 was considered significant. We used the IBM SPSS Statistics 21 software for statistical analysis.

## 3 RESULTS

### 3.1 Low‐altitude simulation treatment significantly improved blood flow and function of the ischemic limb after femoral artery ligation

**FIGURE 1:**
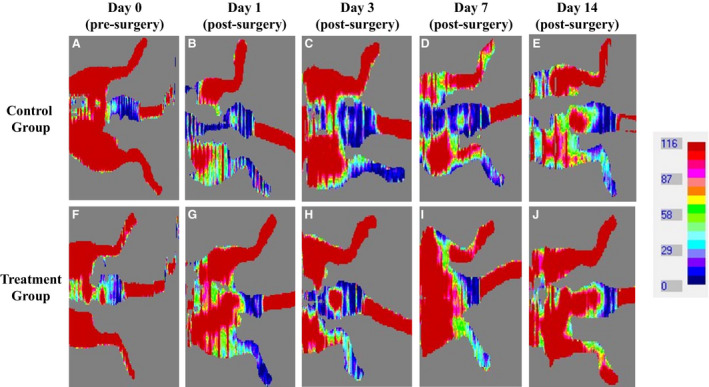
Laser Doppler images reveal that treatment group mice showed a significantly greater improvement in blood perfusion in the intervention limb over the course of 14 days compared to the control group mice [e,j]. Low‐altitude simulation related to reduction in barometric pressure from 754 mmHg to 714 mmHg significantly augments the blood flow in the ischemic hindlimbs of mice

**FIGURE 2:**
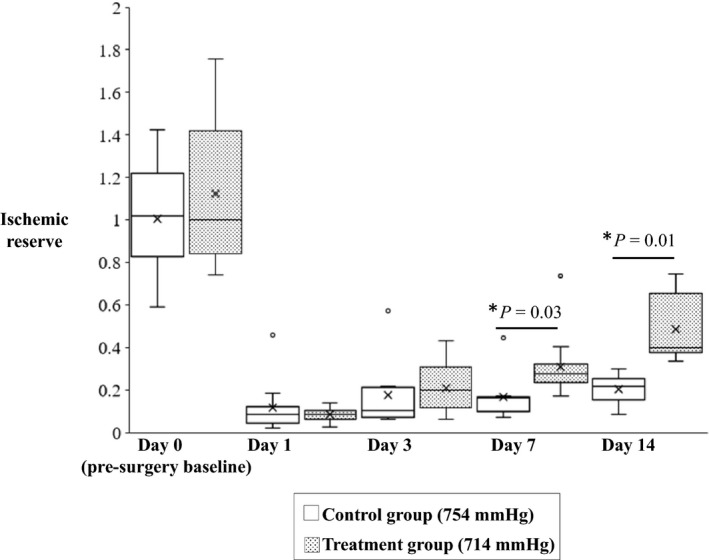
Quantitative analysis of the laser Doppler image data shows improved blood perfusion in the ischemic limb of animals that received 14 days of low‐altitude simulation at 714 mmHg treatment versus animals that did not. Significant differences in blood perfusion between the treatment group and control group are observed at Day 7 (0.31 ± 0.08 vs. 0.16 ± 0.05; *p* = 0.03) after the surgery and Day 14 (0.50 ± 0.13 vs. 0.20 ± 0.06; *p* = 0.01) after the surgery. Values shown are means ± *SE*

**FIGURE 3:**
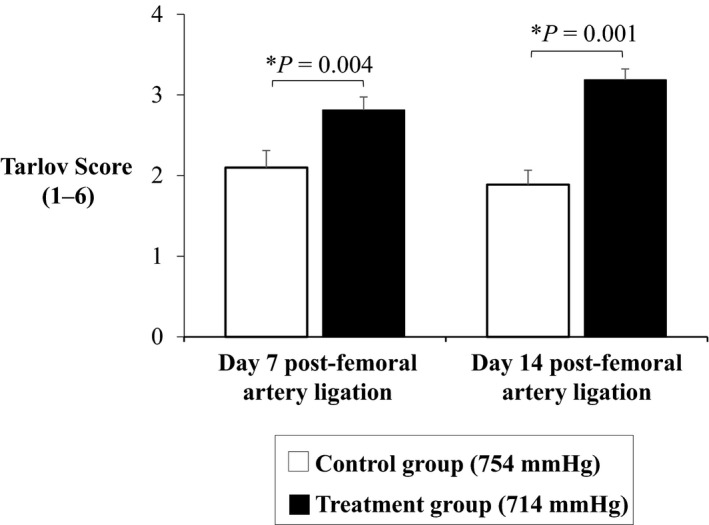
Tarlov scoring showed improved limb function and ability to walk in treatment mice at two weeks versus control mice (3.19 ± 0.14 vs. 1.89 ± 0.18; *p* = 0.001). Values shown are means ± *SE*

After femoral ligation surgery animals were assigned to either 14 days of normoxic low altitude treatment in the hypobaric pressure chamber or no treatment. Laser Doppler images obtained immediately after the surgery showed a clear reduction in blood flow (blue in color) versus the unaffected limb (red in color) (Figure 1b,g). Upon repetition of the laser Doppler imaging after 3, 7, and 14 days, the control animals that were left to recover at room barometric pressure of 754 mmHg showed minor improvement in perfusion of the ischemic hindlimb (Figure 1c–e). However, animals treated with low‐altitude simulation at 714 mmHg for a period of 14 days after the femoral ligation surgery showed a very clear and significant improvement in blood perfusion to the surgically affected limb (Figure 1h–j). Ischemic reserve values generated by normalizing the blood flow of the surgically generated ischemic limb versus the non‐ischemic limb showed statistically significant improvements the blood perfusion in the ischemic hindlimbs of the treatment group animals versus the control group animals at Day 14 (0.5 ± 0.3 vs. 0.2 ± 0.1; *p* = 0.01) (Figure 2). Tarlov scoring showed significant ambulatory improvement in the mice that received normoxic low‐altitude simulation for two weeks in comparison to control mice (3.19 ± 0.14 vs. 1.89 ± 0.18; *p* = 0.001) (Figure 3).

### 3.2 Normoxic low‐altitude simulation treatment was associated with higher lectin perfusion of calf tissue in the ischemic limbs of treatment mice

**FIGURE 4:**
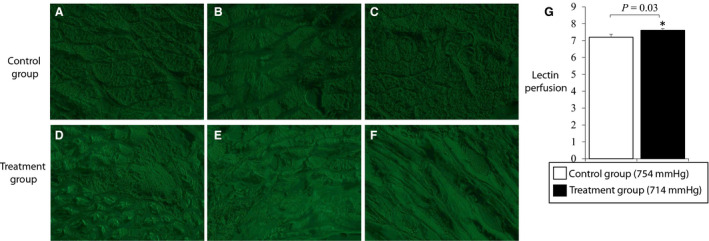
Lectin perfusion staining shows a small but statistically significant improvement of lectin signal in calf tissue extracted from the ischemic limb of normoxic low‐altitude simulation in treatment animals (a–c) versus control animals (d–f) (7.61 ± 0.10 vs. 7.20 ± 0.17; *p* = 0.03). The ischemic reserve is quantified and represented in Figure 3g. Values shown are means ± *SE*.

To evaluate whether greater blood perfusion with normoxic low altitude treatment was associated with enhanced neovascularization, we evaluated microvascular density of blood vessels using fluorescence microscopy of lectin‐perfused ischemic hindlimb tissues. Fourteen days after femoral ligation surgery, lectin was injected into select animals before the excision of calf muscle in the surgical limb for lectin perfusion analysis. The analysis showed small but significant differences in the lectin signal (as measured by green signal) in the calf tissue of animals that did not receive low‐altitude simulation (Figure 4a–c] versus treatment animals that received 14 days of normoxic low‐altitude simulation treatment at 714 mmHg (Figure 4d–f). The lectin ischemic reserve, an index of small blood vessel density (tissue vascularity), was improved in the treatment group mice versus the control group (7.61 ± 0.10 vs. 7.20 ± 0.17; *p* = 0.03) (Figure 4g).

### 3.3 Normoxic low‐altitude simulation treatment reduced HIF‐1α expression in the calf tissue of mice after femoral ligation surgery

**FIGURE 5:**
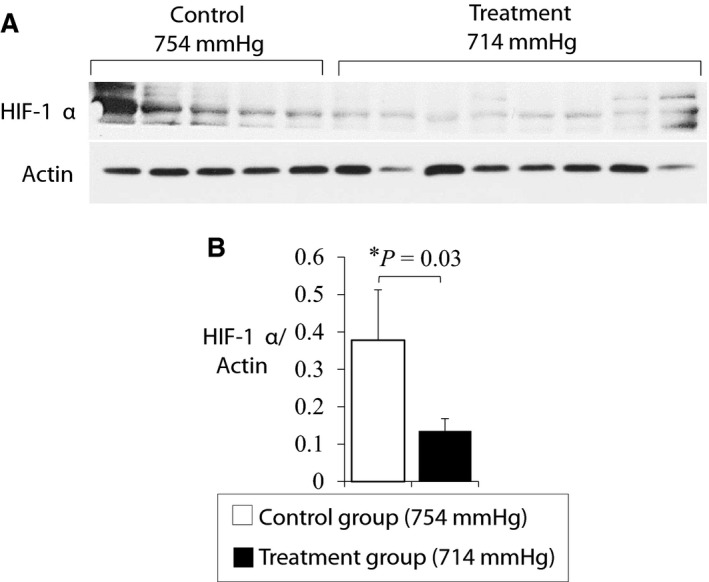
Western blotting showed a decrease in HIF1α expression in calf tissue obtained from treatment animals versus control animals [a,b] (0.13 ± 0.03 vs. 0.37 ± 0.13; *p* = 0.03). The values were normalized to actin to control for loading differences and analyzed with ImageJ Software. Values shown are means ± *SE*

To evaluate whether observed changes in blood perfusion could be related to differences in oxygen tension, we measured the expression of HIF‐1α which is increased under hypoxic or ischemic conditions. Western blot analysis of calf tissue excised from select animals after Day 14 of the study showed a significantly reduced expression of HIF‐1α in animals that received normoxic low‐altitude simulation treatment (at 714 mmHg) for 14 days after femoral artery ligation surgery in comparison to control animals (0.13 ± 0.03 vs. 0.37 ± 0.13; *p* = 0.03) (Figure 5a,b). All bands were normalized to actin expression to correct for loading differences in the gel.

## 4 DISCUSSION

**FIGURE 6:**
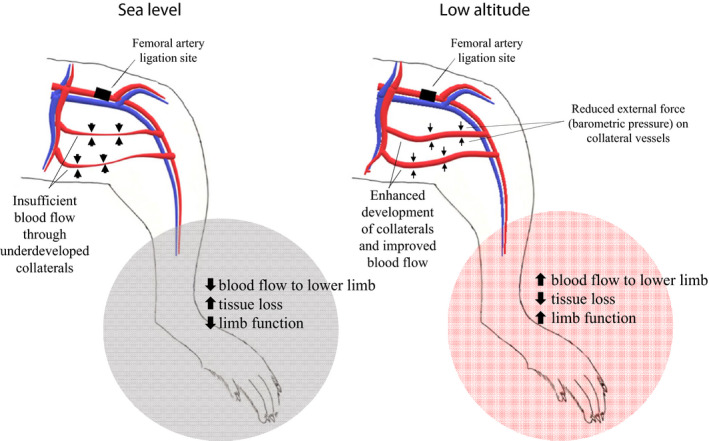
Reduced barometric pressure at low altitudes may improve blood perfusion to the lower limb after femoral artery ligation by enhancing the development of collateral vessels, reducing tissue loss and improving function in the affected limb

We performed a set of experiments to evaluate how low‐altitude simulation could influence lower limb blood perfusion in a murine model of PAD. Our most significant finding is the ability of normoxic low‐altitude simulation to significantly improve blood flow perfusion in mouse models of HLI over a short period of 14 days. A decreased expression of HIF1α in the calf tissue of treatment mice after exposure to low altitude shows that the benefit of the treatment is not due to a downstream effect of hypoxia. We have previously shown a similar therapeutic benefit in a mouse model of MI (Shahid et al., 2019) and believe a mechanical mechanism where reductions in barometric pressure reduce the external compressive forces on arteries, thereby improving vessel dilation and blood flow to ischemic areas is likely (Figure 6).

We achieved a twofold increase in blood perfusion of ischemic hindlimbs of treatment group mice versus control mice by acutely simulating low altitude. This finding was accompanied by a significant functional improvement in the treatment group mice, who were able to walk and use their affected hindlimb visibly better than their control group counterparts. Enhanced perfusion and function achieved by reductions in barometric pressure has not been reported before in laboratory settings. Therapies that aim to increase angiogenesis and vasculogenesis under ischemic conditions have been considered a promising path to finding new and novel therapies for PAD. Physical agents have been used to improve hindlimb ischemia in a comparable manner. Acute thermal therapy (heat) through use of a far‐infrared dry sauna at 41°C followed by 34°C has been used to increase the ischemic limb/normal side blood perfusion ratio in hindlimb ischemia models and an upregulation of eNOS in treatment animals has been cited as the reason behind the noted improvement (Akasaki et al., 2006). Nitric oxide is a major endogenous vasodilator (Jones & Bolli, 2006) and could potentially improve blood perfusion in a model of HLI through vasodilation of the vasculature. By achieving mechanical distension of peripheral vasculature by small reductions in barometric pressure as would be seen with a slight increase in altitude, we can lessen the effects of HLI.

Low energy shockwave treatment has also been used to induce angiogenesis through VEGF receptor 2 simulation therapy to provide relief to ischemic muscles in models of HLI (Holfeld et al., 2014). Laser Doppler imaging of these animals showed a significant improvement in the lower limb blood perfusion of the treatment mice. To increase gastrocnemius muscle angiogenesis in HLI mice, a 2010 study used non‐invasive electroporation with fibroblast growth factor‐2 (FGF‐2) delivery to the ischemic issue post femoral artery ligation. This study found a large improvement in the blood perfusion of the ischemic muscle (Ferraro et al., 2010).

We report a small but statistically significant increase in lectin density in the calf muscle tissue of animals that receive low altitude treatment. Increased lectin density is a hallmark of new blood vessel formation in the area of interest (Grazul‐Bilska et al., 2013). Angiogenesis is a notable downstream effect of hypoxia, regulated by upregulation of HIF‐1α in ischemic tissues (Hashimoto & Shibasaki, 2015). We report a reduction in the expression of HIF‐1α in the treatment group animals after low‐altitude simulation, and as HIF‐1α is a major regulator of angiogenic factors such as VEGF, we do not expect any changes in VEGF after low altitude exposure in our treatment animals. We believe that a mechanical effect of reduction in barometric pressure is reduction of external compressive forces on the systemic vasculature. This may have the effect of improving blood flow through collateral vessels and simulate arteriogenesis and lead to a functional improvement as we demonstrated. Whether arteriogenesis alone, or together with angiogenesis improves blood flow with low‐altitude simulation is unclear and warrants further preclinical work.

### 4.1 Limitations and strengths

A limitation to our study is the lack of measurements of oxygen partial pressure inside our chamber while the animals received treatment. Lowering barometric pressure can reduce the partial pressure of oxygen, however, we suspect this effect would be small at our chosen pressure (714 mmHg) as it mimics a low altitude of 523 m and altitudes below 2000 m are not associated with PO_2_ changes large to cause significant hypoxia in mammalian tissues (Baker, 1969; Cudaback, 1983; Honig, 1988; Zander & Vaupel, 1985).

Additionally, laser Doppler imaging struggles with a few limitations worth noting. It has been well documented that laser Doppler imaging scans and generated color coded numerical values of blood perfusion can be influenced rather heavily by small changes in ambient lighting, animal body temperature, inappropriately removed fur from the skin being imaged, distance of the subject from the scanner, and changes in levels of inhaled anesthesia like isoflurane (Greco et al., 2013). To make sure that our measurements were as accurate as possible, careful consideration was used to keep these variables as consistent as possible across measurements taken at different time points. In addition, as our experiments evaluated acute responses, and we cannot predict whether chronic adaptations to low altitude could attenuate or reverse the changes we observe in our study. Significant strengths of our work include identifying a blood perfusion benefit from an easy to implement low‐altitude simulation procedure.

### 4.2 Clinical implications

Our data support that small reductions in barometric pressure that are seen with low elevations can be used to significantly improve blood perfusion in cases of lower limb ischemia. Improvement in blood perfusion of the peripheral vasculature is associated with a lower risk for MI and stroke in humans and this could be reason behind the epidemiologic phenomenon that people living at higher elevations have a lower risk for cardiovascular diseases. The main therapeutic approach to ischemic disorders including PAD involves improving blood flow to ischemic tissues through exercise or other more rigorous methods. A non‐invasive mechanical technique of reducing barometric pressure to achieve better blood perfusion may become a valuable therapeutic tool for treating conditions like PAD. Furthermore, altitude simulation is a fairly cost effective and easy‐to‐implement means of treatment, occurring in passenger aircraft (Cottrell, 1988; Humphreys et al., 2005), and negative pressure hospital rooms (Nicas et al., 1993) every day.

To conclude, we show that we can enhance lower limb perfusion and improve limb function in mice with hindlimb ischemia with an acute application of low‐altitude simulation mimicking 523 m. These effects appear independent of HIF1α activity as may be seen with hypoxia and may have a potential therapeutic benefit in treating the symptoms of peripheral arterial disease.

## CONFLICT OF INTEREST

There are no competing interests declared for any of the authors.

## Supporting information



Figure S1
