# Supplementary figures and images for: Normoxic low‐altitude simulation (at 714 mmHg) improves limb blood perfusion in mice with hindlimb ischemia
Source: Physiol Rep. 2021 Jan 27;9(2):e14228. doi: 10.14814/phy2.14228 (PMC7839326; doi:10.14814/phy2.14228)

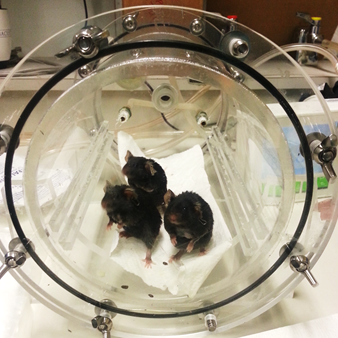

Supplement: Supplementary file 1 — Figure S1 [file PHY2-9-e14228-s001.PNG]
